# Kardiale MRT bei nichtischämischen Kardiomyopathien

**DOI:** 10.1007/s00117-022-01068-6

**Published:** 2022-09-21

**Authors:** Christian Lücke, Matthias Gutberlet

**Affiliations:** 1 Ltd. OA MRT Radiologie, Abteilung für Diagnostische und Interventionelle Radiologie, Herzzentrum Leipzig, Strümpellstr. 39, 04289 Leipzig, Deutschland; 2 Leiter Abteilung für Diagnostische und Interventionelle Radiologie, Professur für Kardiologische Bildgebung in der Radiologie der Universität Leipzig, Herzzentrum Leipzig, Strümpellstr. 39, 04289 Leipzig, Deutschland

**Keywords:** Dilatative Kardiomyopathie, Hypertrophe Kardiomyopathie, Restriktive Kardiomyopathie, Arrhythmogene rechtsventrikuläre Kardiomyopathie, Magnetresonanztomographie, Dilated cardiomyopathy, Hypertrophic cardiomyopathy, Restrictive cardiomyopathy, Arrhythmogenic right ventricular cardiomyopathy, Magnetic resonance imaging

## Abstract

**Hintergrund:**

Die in Deutschland angewandte Einteilung der Kardiomyopathien geht auf die Klassifikation der Europäischen Gesellschaft für Kardiologie (ESC) von 2008 zurück. Dort werden sie nach ihrem Phänotyp unterteilt, so dass die Magnetresonanztomographie (MRT) in der Lage ist, die unterschiedlichen Kardiomyopathien zu differenzieren.

**Bildgebung und Differenzialdiagnostik:**

Die Stärke der MRT ist es, anhand der Möglichkeiten der Gewebsdifferenzierung nichtischämische Kardiomyopathien von anderen Erkrankungen mit ähnlichen morphofunktionellen Aspekten zu differenzieren. So gelingt im Fall der dilatativen Kardiomyopathie (DCM) eine Differenzierung zur inflammatorischen DCM. Im Fall der hypertrophen Kardiomyopathie (HCM) kann analog zur Echographie eine obstruktive und nichtobstruktive Form differenziert werden, aber auch die Detektion einer Amyloidose oder eines Morbus Fabry ist möglich. Die Evaluation der rechtsventrikulären Funktion gelingt im Rahmen einer arrhythmogenen rechtsventrikulären Kardiomyopathie (ARVC) zuverlässig. Außerdem ist die MRT in der Lage, die charakteristische fettige Ersatzfibrose direkt nachzuweisen. Bei den seltenen restriktiven Kardiomyopathien kann sie die Restriktion nachvollziehen und z. B. mittels T1-, T2- und T2*-Mapping die Sphingolipid-Akkumulation im Myokard bei einem Morbus Fabry oder eine Eisenüberladung bei Hämochromatose nachvollziehen.

**Innovationen:**

Die quantitativen Verfahren des parametrischen Mappings bieten die Möglichkeit eines Therapiemonitorings; die klinische Relevanz dieses Monitorings ist aber noch Gegenstand aktueller Forschung. Die unklassifizierten Kardiomyopathien können sich klinisch mit ähnlicher Symptomatik wie ischämische oder inflammatorische Erkrankungen präsentieren, so dass im Fall eines Myokardinfarkts ohne verschlossene Koronararterien („myocardial infarction without obstructive coronary arteries“, MINOCA) in der Herzkatheteruntersuchung die MRT ein entscheidendes diagnostisches Instrument ist, um die tatsächlich zugrundeliegende Erkrankung festzustellen. Gleichermaßen kann sie bei neuen Kardiomyopathien wie der Non-compaction-Kardiomyopathie der Wegbereiter für eine morphologische Krankheitsdefinition sein.

## Abstract (en)

**Background:**

The classification of cardiomyopathies used in Germany goes back to the European Society of Cardiology (ESC) classification of 2008. The cardiomyopathies are subdivided according to the phenotype, so that magnetic resonance imaging (MRI) is able to differentiate between the various cardiomyopathies.

**Imaging and differential diagnostics:**

The strength of MRI is the ability to differentiate nonischemic cardiomyopathies from other diseases with similar morphofunctional aspects, based on the possibilities of tissue differentiation. In the case of dilated cardiomyopathy (DCM), for example, a differentiation from inflammatory DCM is possible. In the case of hypertrophic cardiomyopathy (HCM), obstructive and nonobstructive forms can be differentiated analogously to the echo but amyloidosis or Fabry disease can also be detected. Evaluation of the right ventricular function is reliable in arrhythmogenic right ventricular cardiomyopathy (ARVC). The use of MRI is also able to directly detect the characteristic fibrofatty degeneration. In the rare restrictive cardiomyopathies (RCM), MRI can track restriction and, for example by means of T1, T2 and T2* mapping, detect sphingolipid accumulation in the myocardium in the context of Fabry disease or iron overload in the context of hemochromatosis.

**Innovations:**

The quantitative methods of parametric mapping provide the possibility of treatment monitoring but the clinical relevance of this monitoring is still the subject of current research. The unclassified cardiomyopathies can present clinically with similar symptoms to ischemic or inflammatory diseases, so that in the case of myocardial infarction without obstructive coronary arteries (MINOCA) in cardiac catheterization, MRI is a decisive diagnostic tool to determine the actual underlying disease. Similarly, in new cardiomyopathies such as noncompaction cardiomyopathy, MRI can pave the way for a morphological disease definition.

Die in Deutschland aktuell angewandte Klassifikation der Kardiomyopathien geht auf das Positionspapier der Europäischen Gesellschaft für Kardiologie (ESC) (Abb. 1) aus dem Jahr 2008 [1] und auf die Klassifikation der American Heart Association (AHA) von 2006 [2] zurück (Abb. 2).

**Figure 1:**
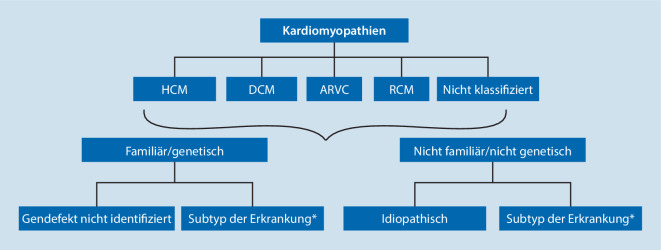

**Figure 2:**
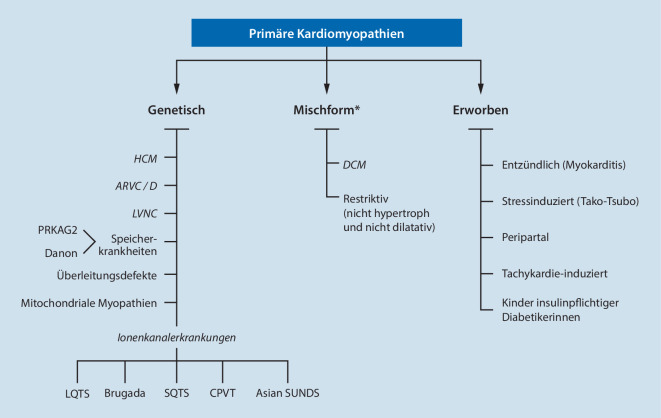

Die Klassifikation der ESC stützt sich im Wesentlichen auf den Phänotyp mit einer sekundären Unterscheidung in eine familiäre/genetische Form oder eine nichtfamiliäre/nichtgenetische Form (Abb. 2).

In der amerikanischen Klassifikation werden vorrangig nur die primären Kardiomyopathien klassifiziert, in denen insbesondere das Herz das erkrankte Organ ist, und so von sekundären Kardiomyopathien unterschieden, bei denen eine systemische Erkrankung vorliegt und das Herz sekundär betroffen ist. In einer weiteren Subtypisierung werden genetische von erworbenen Formen unterschieden, aber auch gemischte Formen beschrieben. Die AHA-Klassifikation beinhaltet aber auch die Ionenkanalerkrankungen, bei denen sich der Herzmuskel selbst nicht bildmorphologisch-phänotypisch verändert darstellt und die Bildgebung deshalb bisher keine Rolle spielt.

Alle verwendeten Klassifikationen gehen aber auf die Definition der Kardiomyopathien der Weltgesundheitsorganisation (WHO) von 1980 [3] zurück, die Kardiomyopathien als *Herzmuskelerkrankungen unbekannter Ursache* definieren, um sie gegenüber Herzmuskelerkrankungen bekannter Ursache (Bluthochdruck, koronare Herzerkrankung, Klappenerkrankungen etc.) abzugrenzen.

Neue wissenschaftliche Erkenntnisse und neue Untersuchungsmethoden haben immer wieder zu Modifikationen der Klassifikationen geführt. Die grundsätzliche Klassifikation der ESC von 2008 und der AHA von 2006 wurden aber in der letzten Dekade nicht mehr verändert. Morphologische und funktionelle Veränderungen können zu einer unterschiedlichen Einordnung führen. So kann z. B. bei einer kardialen Amyloidose der linke Ventrikel bildmorphologisch wie eine Hypertrophie imponieren, aber funktionell auch ein restriktives Füllungsmuster aufweisen. Solche Subtypen machten dann immer wieder eine Reklassifizierung notwendig.

In der klinischen Routine drückt sich diese Unschärfe der Klassifizierung insbesondere auch dadurch aus, dass Begriffe wie die *ischämische Kardiomyopathie* oder *hypertensive Kardiomyopathie* verwendet werden. Des Weiteren werden manche Kardiomyopathien auch nach anamnestischen Kriterien definiert, wie die peripartale Kardiomyopathie, deren Krankheitsursache bisher nicht eindeutig geklärt ist und den Phänotyp einer dilatativen Kardiomyopathie aufweist.

## Hypertrophe Kardiomyopathien

Die hypertrophe Kardiomyopathie ist die häufigste genetische kardiovaskuläre Erkrankung mit einer Inzidenz von 1 in 200–300 Fällen [4]. Sie basiert nach morphologischen Kriterien auf dem Phänotyp einer linksventrikulären Hypertrophie und dem Fehlen einer Ursache [5, 6]. In der klinischen Routine besteht die Notwendigkeit, gegenüber erworbenen kardialen Erkrankungen zu differenzieren [7, 8]. So kann ein unbehandelter Hypertonus ebenso [9, 10] wie eine Aortenstenose oder eine Speichererkrankung, z. B. eine kardiale Amyloidose [11], Ursache einer Hypertrophie sein.

**Figure 3:**
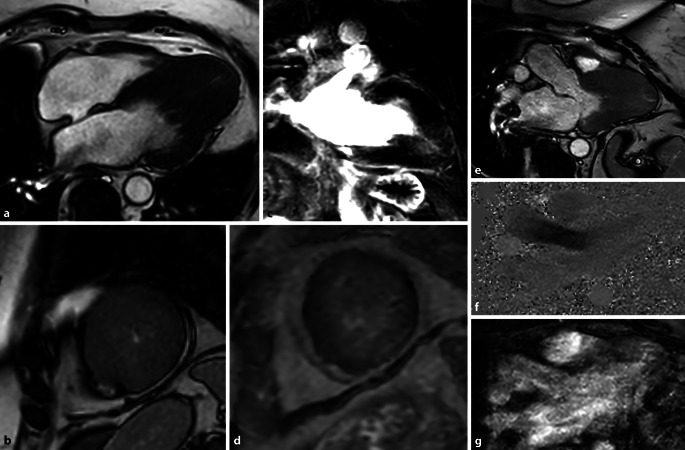

Bei den hypertrophen Kardiomyopathien wird, in Abhängigkeit vom Vorhandensein einer Obstruktion des linksventrikulären Ausflusstrakts (LVOT), eine obstruktive (HOCM) von einer nichtobstruktiven Form (HNOCM) unterschieden (Abb. 3).

Die Obstruktion des LVOT wird zunächst visuell beurteilt und dann, meist besser objektivierbar, auch funktionell mittels der Doppler-Echokardiographie oder seltener der Phasenkontrastflussmessung in der MRT (Abb. 3f, g). In den Cine-Sequenzen sieht man durch die Flussbeschleunigung Dephasierungsartefakte im LVOT und eventuell eine systolische Vorwärtsbewegung des anterioren Mitralklappensegels, im Englischen „systolic anterior movement“ oder SAM-Phänomen genannt.

Mittels MRT kann die linksventrikuläre Muskelmasse, als negativ prognostischer Marker, am zuverlässigsten bestimmt werden [13]. Des Weiteren kann über das *Late-Gadolinium-Enhancement* (LGE), das T1-Mapping und eine ECV-Bestimmung die Fibroselast quantifiziert werden [14]. Das LGE zeigt typischerweise ein intramyokardiales, flächig bzw. *patchworkartig* angeordnetes Muster, insbesondere in Bereichen der stärksten Hypertrophie. Dort kann es aufgrund regionaler Ischämien zu fokalen Nekrosen und damit nachfolgend zur Fibrose- und Narbenbildung kommen. Auch ein muskuläres „disarray“ wird als Ursache diskutiert.

## Dilatative Kardiomyopathien

**Figure 4:**
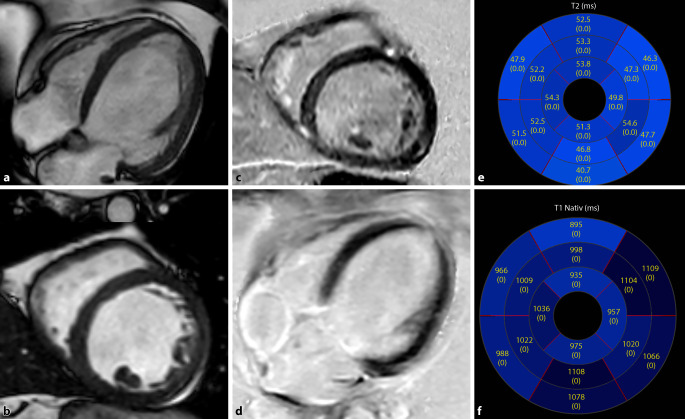

Die dilatative Kardiomyopathie (DCM), charakterisiert durch eine linksventrikuläre Dilatation bei eingeschränkter kardialer Funktion und bei gleichzeitigem Fehlen einer signifikanten koronaren Herzerkrankung, tritt sowohl primär als genetische Erkrankung auf, kann aber auch sekundär im Rahmen von anderen strukturellen Erkrankungen, peripartal oder als Endstadium einer Myokarditis in Form der inflammatorischen DCM auftreten. Sie muss von der ischämischen Kardiomyopathie unterschieden werden. Die myokardiale Gesamtmuskelmasse des linken Ventrikels ist häufig erhöht, auch wenn sich die Wand selbst eher als ausgedünnt bis normal darstellt. Das typische LGE-Muster ist ein sogenanntes „mid-wall-enhancement“ des Septums, welches sich intramyokardial unter Aussparung der endokardialen und epikardialen Anteile darstellt. Diese Form kann aber auch im Rahmen einer inflammatorischen DCM auftreten, deren typische LGE-Muster eher ein *subepikardiales* LGE darstellt. Eine Myokardcharakterisierung mittels Mapping kann bei diesen Patienten grundsätzlich durchgeführt werden, sich aufgrund der dünnen Wand aber als schwierig gestalten ([14, 15]; Abb. 4).

## Restriktive Kardiomyopathien

Die restriktive Kardiomyopathie (RCM) ist die seltenste Form der Kardiomyopathien. Sie ist als ein restriktives Füllungsmuster bei einem reduzierten enddiastolischen Volumen eines oder beider Ventrikel mit erhaltener systolischer Funktion und normaler oder gering erhöhter Wanddicke definiert [16]. Da sie häufig sekundär aufgrund von systemischen Erkrankungen auftritt, gibt es viele Überschneidungen mit dem Begriff der infiltrativen Kardiomyopathie bzw. von Speichererkrankungen. Auch die HCM kann ein restriktives Füllungsmuster aufweisen. Aufgrund der erhöhten myokardialen Steifigkeit führt eine Volumenbelastung zu einem steilen Anstieg des intraventrikulären linksventrikulären (LV) Drucks, was mittels Druckvolumenkurven im Rahmen einer Herzkatheteruntersuchung gemessen werden kann. Es werden primäre familiäre von sekundären Formen unterschieden, von denen hier nur einzelne exemplarisch (*kursiv markiert*) dargestellt werden sollen: [16]:

1. *Amyloidose (Leichtketten-Amyloidose [AL], Transthyretin-Amyloidose [ATTR])*
2. *Sarkoidose*
3. Hämochromatose
4. *Eosinophile myokardiale Erkrankungen (Löffler-Endokarditis)*
5. Idiopathische restriktive Kardiomyopathie (RCM)
6. Progressive systemische Sklerose
7. Postradiogene RCM (nach Morbus Hodgkin, Brustkrebs etc.)
8. *Anderson-Fabry-Erkrankung*
9. Danon-Erkrankung
10. Friedreich-Ataxie
11. Diabetische Kardiomyopathie
12. Medikamenteninduzierte RCM
13. Mukopolysaccharidosen
14. Wegener-Granulomatose
15. Kardiale Metastasen

## Amyloidose

**Figure 5:**
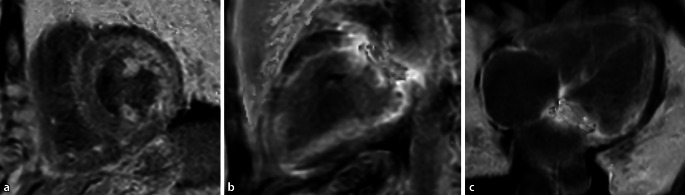

Die kardiale Amyloidose gilt als die am schnellsten fortschreitende Form von Herzerkrankungen mit einem medianen Überleben bei unbehandelter Erkrankung von < 6 Monaten bei der Leichtketten-Amyloidose bis hin zu 3–5 Jahren für die ATTR-Amyloidose [17]. Sie geht sehr häufig mit einer Hypertrophie des Myokards einher und stellt eine wichtige Differenzialdiagnose in der Ursachenfindung einer kardialen Hypertrophie dar. Neben der initialen Echokardiographie liefert die kardiale MRT zusätzliche Informationen über die Myokardbeschaffenheit, und obgleich eine geringe Erhöhung des Extrazellularvolumens (ECV) auch bei anderen Erkrankungen im Rahmen einer diffusen Fibrose oder eines Ödems auftritt, ist eine massive ECV-Vergrößerung schon nahezu pathognomonisch für eine kardiale Amyloidose mit ausgeprägter interstitieller Ablagerung des Amyloids (Abb. 5).

Nuklearmedizinische Verfahren sind sehr sensitiv für eine ATTR (Tc-99m-DPD, Tc-99m-PYP, or Tc-99m-HMDP-Szintigraphie) und neuere Amyloid-Tracer können in einer Positronen-Emissions-Tomographie (PET) ebenfalls eine kardiale Amyloidose zuverlässig nachweisen [18].

Die myokardiale Amyloidose kann in der MRT gut mit myokardialem Mapping diagnostiziert werden. Aufgrund der interstitiellen Amyloidablagerungen und der dadurch deutlich verlängerten T1-Zeit gelingt dies sogar in nicht kontrastverstärktem T1-Mapping [8, 19].

**Figure 6:**
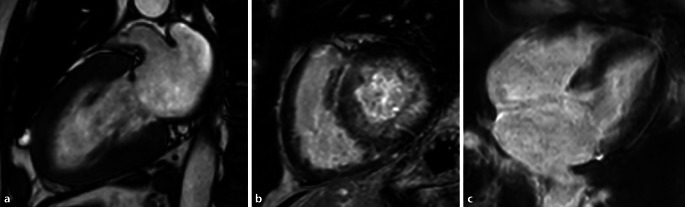

Dabei können sowohl die AL- als auch die ATTR-Amyloidose nachgewiesen werden ([20]; Abb. 6).

Durch Kontrastmittelgabe zeigen die extrazellulären Amyloidablagerungen ein MR-tomographisch gut detektierbares LGE-Muster und in der ECV-Analyse einen deutlich vergrößerten Extrazellularraum [21]. Dabei zeigt die ECV-Bestimmung eine bessere diagnostische Genauigkeit im Vergleich zum klassischen LGE oder dem nativen T1-Mapping [22]. T1-Zeiten und ECV sind bei der ATTR-Amyloidose deutlich verlängert und in Studien meist etwas niedriger als bei der AL-Amyloidose. Scoringsysteme zur Differenzierung existieren, die Differenzierung allein durch die MRT bleibt jedoch im Vergleich zum Referenzstandard Biopsie schwierig.

Es kann korrespondierend zur Echographie mittels MRT ein basoapikaler Gradient der Funktionseinschränkung in Korrelation mit erhöhten T1-Zeiten beobachtet werden, die von basal nach apikal abnimmt [23]. Auch mit der Computertomographie (CT) ist eine ECV-Bestimmung grundsätzlich möglich, wenn ein Nativ-Scan und ein Equilibrium-Scan nach 5 min durchgeführt werden [24]. Aber auch die posttherapeutische Verringerung der LV-Masse, z. B. nach Gabe von Grüntee-Extrakt (Epigallocatechin-3-Galleat), und somit die posttherapeutische Verlaufskontrolle einer ATTR-Amyloidose lässt sich mittels T1-Mapping und einer ECV-Analyse durchführen [25].

Sowohl mittels MRT als auch PET gelingt die zuverlässige Detektion der Amyloidose und Differenzierung gegenüber anderen Kardiomyopathien. In der Subklassifizierung von AL- und ATTR-Amyloidose scheint aber die Szintigraphie überlegen zu sein [26]. Interessant dürfte die zeitgleiche Analyse in Hybrid-PET/MR-Tomographen sein, sofern sie verfügbar sind [18, 27].

## Sarkoidose

**Figure 7:**
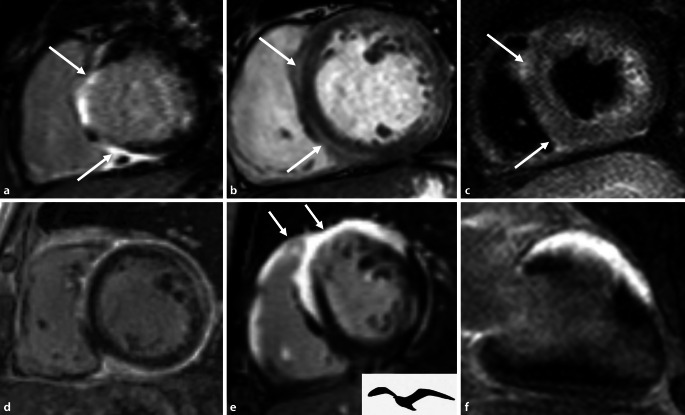

Die Sarkoidose ist eine multisystemische granulomatös-entzündliche Erkrankung, die am häufigsten die Lunge und Lymphknoten befällt, sich aber auch als neurologische oder kardiale Erkrankung äußern kann. Ein spezifischer Test besteht für die Erkrankung nicht, sodass die Erkrankung eine Ausschlussdiagnose bleibt [28]. Sie weist eine von der Herkunft abhängige, regional unterschiedliche Inzidenz (bei Japanern 1–2/100.000) mit starkem Nord-Süd-Gefälle innerhalb Europas auf (5–60/100.000), die innerhalb Skandinaviens am höchsten ist [29, 30]. Eine klinisch auffällige kardiale Beteiligung, tritt nur bei einem geringen Prozentsatz (1–7 %) der Patienten auf, in Obduktionsstudien findet sich allerdings eine kardiale Beteiligung in bis zu 19,5–78 % der Fälle [31]. Die Bedeutung der kardialen Beteiligung wird unterschiedlich gewertet und reicht von 2 % bis hin zu 85 % als Todesursache [31]. Die Daten bezüglich einer isolierten kardialen Sarkoidose sind spärlich, da auch bei Patienten mit primärem klinischem Verdacht auf eine kardiale Sarkoidose, sich retrospektiv in ca. 40 % der Fälle Zeichen einer pulmonalen Sarkoidose im Röntgenbild finden [32, 33]. Bei der kardialen Sarkoidose tritt auch häufig ein subepikardiales LGE auf wie bei der Myokarditis, dieses ist aber häufig noch ausgeprägter mit epikardialen Granulomen und auch einer rechtsventrikulären Beteiligung. Charakteristisch soll vor allem das „*hug sign*“ sein mit intramyokardialem, septalem LGE, nahe des rechten Ventrikels (RV) im Bereich der Insertionsstellen der freien Wand des RV (Abb. 7a–d) und das *Möwenzeichen* mit LGE im Bereich der anterioren Wand des rechten und linken Ventrikels unter Einbeziehung des interventrikulären Septums (Abb. 7d, e).

## Löffler-Endokarditis, Endomyokardfibrose

**Figure 8:**
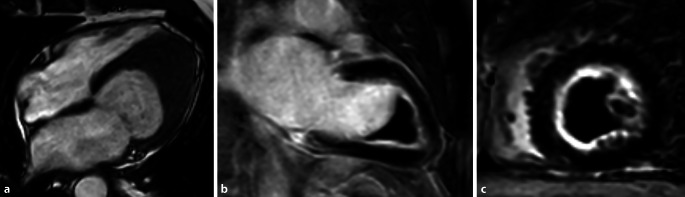

Die seltene Löffler-Endokarditis beschreibt eine akute Form einer restriktiven Kardiomyopathie, die mit einer Eosinophilie einhergeht und eine schlechte Prognose hat. Chronifiziert diese Erkrankung, so spricht man von einer Endomyokardfibrose. Diese Erkrankungen treten insbesondere in tropischen und subtropischen Regionen auf. Durch die ausgeprägte Fibrose des Endokards kommt es zu einer schweren Restriktion, die zu einer beeinträchtigten Füllung, Klappeninsuffizienzen und häufig auch apikaler Thrombenbildung führt. [34]. Ein chirurgischer Eingriff kann unter den richtigen Voraussetzungen das Überleben verbessern (Abb. 8).

## Arrhythmogene rechtsventrikuläre Kardiomyopathie

**Figure 9:**
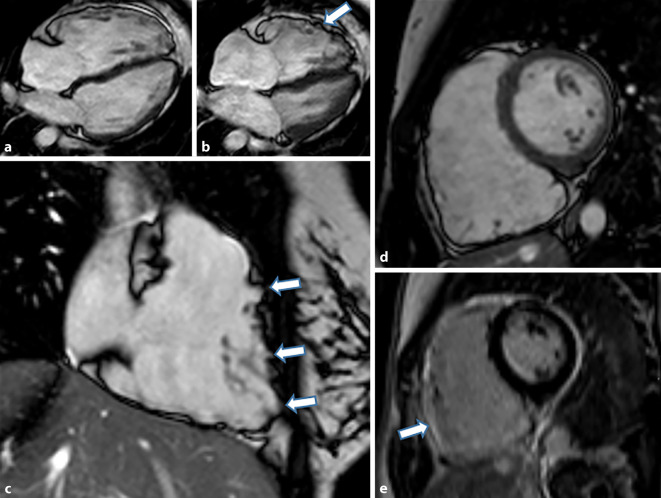

Mit dem Begriff der arrhythmogenen rechtsventrikulären Kardiomyopathie (ARVC; Abb. 9) wird eine Kardiomyopathie beschrieben, die vorrangig den rechten Ventrikel betrifft und sich mit malignen ventrikulären Tachykardien klinisch manifestieren kann [35]. Durch pathologische und histologische Analysen wurde klar, dass ein fibröser fettiger Umbau – vor allem der freien Wand des rechten Ventrikels – das morphologisch-phänotypische Charakteristikum dieser Erkrankung ist, aber auch den linken Ventrikel betreffen kann. Deshalb sollte man diese Erkrankung besser als arrhythmogene Kardiomyopathie (ACM) bezeichnen [35]. Die neuesten Padua-Kriterien aus dem Jahr 2020 fügen auch Kriterien für die linksventrikuläre Form hinzu und betonen die Rolle der Gewebscharakterisierung mittels MRT im Vergleich zu der vorangegangenen Version der Task-Force-Kriterien von 2010, in der das LGE noch nicht als Majorkriterium herangezogen wurde [36].

Die Kriterien umfassen 6 Kategorien (morphologisch-funktionelle und strukturell-myokardiale des rechten oder linken Ventrikels sowie Repolarisierungs‑/Depolarisierungsstörungen, die Familienanamnese und die Genetik), wobei nur die ersten beiden Kategorien mittels Bildgebung erfasst werden können (Tab. 1). Sie gliedern sich in Major- und Minorkriterien.

**Table 1:** 

Majorkriterien	a. Regionale RV-Akinesie, Dyskinesie oder Bulging *plus* *erhöhte RV-EDVI [ml/m*^*2*^*]* und/oder *erniedrigte* RVEF (%) entsprechend altersgematchter Normogramme
	b. Transmurales *LGE („stria pattern“)* von ≥ 1 RV-Region („inlet, outlet, apex“) in 2 orthogonalen Schichten
Minorkriterien	Regionale RV-Akinesie, Dyskinesie oder Bulging *ohne* erhöhten RV-EDVI [ml/m^2^] und/oder erniedrigte RVEF (%)

*RV* rechtsventrikulär, *RVEF* rechtsventrikuläre Ejektionsfraktion, *EDVI* enddiastolischer Volumenindex

Für die rechtsventrikuläre Form (ARVC) muss je nach diagnostischer Sicherheit die entsprechende Anzahl an folgenden Kriterien erfüllt sein, ohne dass eine linksventrikuläre Beteiligung vorliegt:

- *Definitive ARVC:* (2 Major-/1 Major- und 2 Minor- oder 4 Minorkriterien)
- *Borderline-ARVC*: (1 Major- und 1 Minor- oder 4 Minorkriterien)
- *Mögliche ARVC:* (1 Major- oder 2 Minorkriterien unterschiedlicher Kategorien)

Für die biventrikuläre Form muss je mindestens ein morphologisch-funktionelles Kriterium beider Ventrikel vorliegen. Für die linksdominante Form (ALVC) muss neben der strukturellen Abnormalität des LV eine ACM-verursachende Genmutation vorliegen und eine RV-Beteiligung fehlen [35]. Eine scharfe Abgrenzung gegenüber Fettgewebe, welches als normale Komponente des linken Ventrikels oder im Rahmen einer *lipomatösen Metaplasie* nach Myokardschädigung insbesondere nach Myokardinfarkt auftritt, existiert jedoch nicht [37].

## Unklassifizierte Kardiomyopathien

### Tako-Tsubo-Kardiomyopathie (besser Tako-Tsubo-Syndrom)

**Figure 10:**
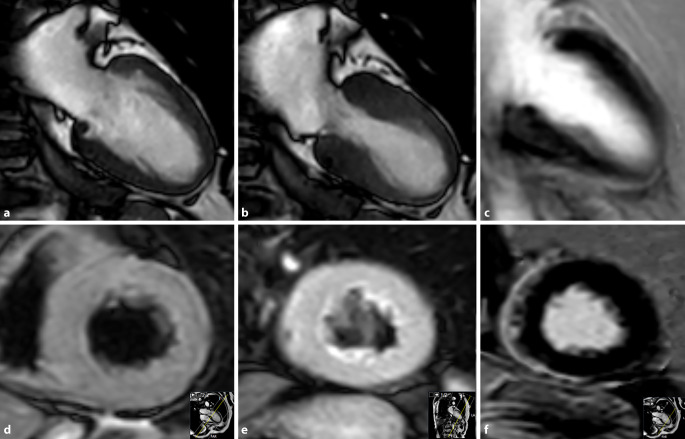

Die nach der japanischen Tintenfischfalle benannte Kardiomyopathie, das Tako-Tsubo-Syndrom (Abb. 10), ist keine Kardiomyopathie im engeren Sinne, wird aber nach den ESC- und AHA-Guidelines noch als solche klassifiziert. Es werden in abnehmender Häufigkeit die typische apikale, mittventrikuläre und basale Form unterschieden, und es gibt auch eine seltene fokale anteriore Form. Es geht häufig ein belastendes psychisches bzw. emotionales Erlebnis voraus, wie z. B. der Verlust des Lebenspartners, weshalb es auch als „*broken heart syndome*“ bezeichnet wird. Die Kinetikstörung kann schnell reversibel (binnen Tagen), aber auch mehrere Wochen vorhanden sein, das fokale Ödem persistiert in der Regel länger (Wochen). Es findet sich typischerweise kein LGE, gelegentlich ist der Extrazellularraum durch das Ödem allerdings so stark erweitert, dass in den LGE-Sequenzen ein (reversibles) diffuses LGE auftreten kann. Im Setting des akuten Koronarsyndroms (ACS) im Fall eines MINOCA („**M**yocardial **I**nfarction with **N**on-**O**bstructive **C**oronary **A**rteries“) besitzt die MRT die Fähigkeit, das Tako-Tsubo-Syndrom mit all seinen Unterformen, von einem Myokardinfarkt eines kleinen Seitenastes oder mit Spontanlyse, sowie einer Myokarditis zu differenzieren [38–40].

## Linksventrikuläre Non-compaction-Kardiomyopathie

**Figure 11:**
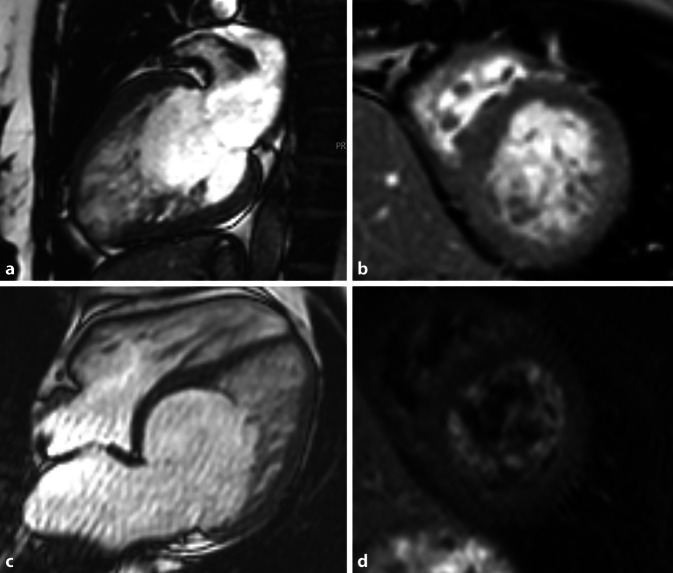

Die linksventrikuläre Non-compaction-Kardiomyopathie (LVNC) ist die zuletzt klassifizierte Kardiomyopathie. Sie ist charakterisiert durch eine abnormale Trabekularisierung des linksventrikulären Myokards bei gleichzeitiger Verminderung des kompaktierten Myokards. Morphologisch können je nach Krankheitsstadium entweder eine linksventrikuläre Dilatation oder nur eine Hypertrophie, funktionell eine diastolische oder systolische Dysfunktion im Vordergrund stehen, oder sie tritt begleitend mit angeborenen Herzfehlern auf [41]. Die gängigste Hypothese über die Ätiologie der LVNC beruht darauf, dass sich das während der Embryogenese zunächst vollständig *trabekularisierte* oder „spongy“ Myokard in einem bestimmten Stadium nicht mehr *kompaktiert*. Eine genetische Veranlagung kann in 30–50 % der Patienten nachgewiesen werden, und mehrere verursachende Gene wurden identifiziert. Die Behandlung einer Herzinsuffizienz sowie die Behandlung von Arrhythmien und die Prävention eines plötzlichen Herztods mittels eines implantierbaren Kardioverter-Defibrillators (ICD) sind die Schwerpunkte der aktuellen Therapie [41]. Die Patienten haben durch die vermehrte Trabekularisierung und den dadurch verlangsamten Blutfluss zwischen den Trabekeln ein etwas erhöhtes Risiko thrombembolischer Ereignisse [42]. Durch eine absolute Quantifizierung der nichtkompaktierten Myokardmasse mittels Planimetrie in Cine-Sequenzen und die Detektion vermehrt trabekularisierten Myokards in den basalen Segmenten im Verhältnis > 3:1 gelingt die sichere Detektion und Differenzierung der LVNC gegenüber anderen Kardiomyopathien [43]. Typischerweise finden sich beim normalen Herzen, vor allem im Bereich der linksventrikulären Seite des interventrikulären Septums, insbesondere basal und mittventrikulär keine Trabekel, was diagnostisch für die LVNC genutzt werden kann (Abb. 11).

### Merke.

Ein LGE tritt bei der LVNC typischerweise nicht auf. Aufgrund der starken Trabekularisierung kann jedoch bei nicht optimal in der Diastole akquirierter LGE-Sequenz zwischen den Trabekeln *gefangenes* KM als intramyokardiales LGE fehlgedeutet werden.

## Inflammatorische Kardiomyopathie und Myokarditis

Die Myokarditis wird auch als das Chamäleon unter den myokardialen Erkrankungen bezeichnet. Typische Symptome der akuten Myokarditis sind Brustschmerz, Dyspnoe, Abgeschlagenheit, Palpitationen, Synkope oder kardiogener Schock. Aufgrund dieser unspezifischen Klinik kann es mitunter schwierig sein, dieses Krankheitsbild gegenüber anderen kardialen Erkrankungen zu differenzieren. Patienten weisen teils Symptome eines Infarkts, einer Herzinsuffizienz, eines akuten Infektionsgeschehens oder auch von Herzrhythmusstörungen bis hin zu einem überlebten plötzlichen Herztod auf [44–47].

Die Ätiologie der Myokarditis ist ebenfalls sehr heterogen: Häufig liegt eine banale virale Infektion vor, ebenso können aber auch bakterielle, parasitäre oder Pilzinfektionen zu einer infektiösen Myokarditis führen. Des Weiteren können toxische Substanzen, Medikamente oder systemische Immunreaktionen eine Myokarditis verursachen [44]. Zuletzt ist dieses Krankheitsbild im Rahmen einer COVID-19-Infektion oder deutlich seltener als Nebenwirkung bei jungen Menschen im Rahmen einer COVID-19-Impfung in den Fokus der Öffentlichkeit gerückt [48].

Ebenso heterogen wie die Ätiologie ist auch die Prognose für Patienten mit dieser Erkrankung. Während sie meist folgenlos ausheilt, kann sie ebenso fulminant verlaufen, reaktiviert werden oder auch in einer inflammatorischen dilatativen Kardiomyopathie (iDCM) münden [44, 47].

Aufgrund der heterogenen Ätiologie und Prognose gestaltet sich die Therapie ebenfalls heterogen und reicht von Empfehlung eines vorübergehenden Sportsverzichts über unspezifische Herzinsuffizienztherapie oder spezifische antivirale Therapie bis hin zur Anwendung von kardialen Unterstützungssystemen und der Herztransplantation [44].

Die WHO-Definition der inflammatorischen Veränderungen des Myokards unterscheidet eine Myokarditis (auf der Basis histologischen, immunologischen und immunhistochemischen Kriterien) von einer inflammatorischen Kardiomyopathie und einer dilatativen Kardiomyopathie [49]. Im klinischen Alltag ist diese Differenzierung in der Regel nicht möglich. Die Endomyokardbiopsie ist der invasive Goldstandard, um eine weitere Differenzierung der Myokarditis zu ermöglichen, wird in der klinischen Routine jedoch nur selten durchgeführt [49]. Es hat sich deshalb ein *klinischer Goldstandard* basierend auf den Symptomen der akuten Myokarditis etabliert [50–52]; spezifische Therapien zeigen bisher keinen eindeutigen Vorteil gegenüber symptomatischer Therapien [53].

Heutzutage ist der nichtinvasive, klinische Goldstandard für den Nachweis einer Myokarditis die MRT auf der Basis der 2018 modifizierten Lake-Louise-Kriterien, bei denen die parametrische Bildgebung eine wesentliche Rolle spielt [54].

Bei Patienten mit klinischen Hinweisen für eine schwere subakute Myokarditis gelang eine Differenzierung gegenüber Gesunden mit einer schrittweisen Herangehensweise unter Anwendung des LGE und des ECV mit einer diagnostischen Genauigkeit von bis zu 90 %, wobei keine bioptische Sicherung erfolgte. Die Diagnose erfolgte allein aufgrund der klinischen Symptomatik. Es stellt sich die Frage, wie notwendig die MRT-Diagnose in der akuten Phase der myokardialen Inflammation ist, wenn sie bereits allein klinisch wie in dieser Studie zu stellen war [55, 56].

**Figure 12:**
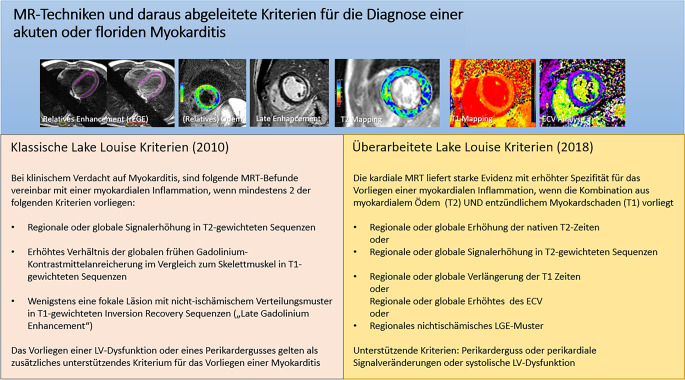

Mit multiparametrischer Bildgebung mittels T1- und T2-Mapping gelingt es Patienten mit und ohne aktive Inflammation in der Myokardbiopsie, die sich mit kürzlich aufgetretener Herzinsuffizienz vorstellen, zu differenzieren, wobei insbesondere die T2-Bildgebung den aktiven Inflammationszustand nachweist ([57]; Abb. 12).

## Fazit für die Praxis

- Die kardiale Magnetresonanztomographie (MRT) hilft, die unterschiedlichen Phänotypen der Kardiomyopathien zu differenzieren, und liefert über die Gewebsdifferenzierung (Ödem und Late-Gadolinium-Enhancement) Differenzierungsmöglichkeiten und prognostische Aussagen für die klinische Praxis.
- Die parametrische Bildgebung (T1-, T2-, T2*-Mapping) hilft dabei, auch seltenere Differenzialdiagnosen zu detektieren und zu quantifizieren sowie den Verlauf zu kontrollieren.
- Die kardiale MRT ist integraler Bestandteil in der Abklärung des akuten Thoraxschmerzes, wenn kein akuter Myokardinfarkt vorliegt (z. B. Myokardinfarkt ohne verschlossene Koronararterien, MINOCA), aber auch zur weiteren Differenzialdiagnostik bei Patienten mit Herzinsuffizienz oder unspezifischen kardialen Symptomen.
